# A Simple Halide-to-Anion Exchange Method for Heteroaromatic Salts and Ionic Liquids

**DOI:** 10.3390/molecules17044007

**Published:** 2012-04-02

**Authors:** Ermitas Alcalde, Immaculada Dinarès, Anna Ibáñez, Neus Mesquida

**Affiliations:** Laboratory of Organic Chemistry, Faculty of Pharmacy, University of Barcelona, Joan XXIII s/n, 08028 Barcelona, Spain; Email: aibaneji7@alumnes.ub.edu (A.I.); neusmesquida@ub.edu (N.M.)

**Keywords:** imidazolium salts, pyridinium salts, ammonium salts, anion exchange resin, counteranion exchange, ionic liquids

## Abstract

A broad and simple method permitted halide ions in quaternary heteroaromatic and ammonium salts to be exchanged for a variety of anions using an anion exchange resin (A^−^ form) in non-aqueous media. The anion loading of the AER (OH^−^ form) was examined using two different anion sources, acids or ammonium salts, and changing the polarity of the solvents. The AER (A^−^ form) method in organic solvents was then applied to several quaternary heteroaromatic salts and ILs, and the anion exchange proceeded in excellent to quantitative yields, concomitantly removing halide impurities. Relying on the hydrophobicity of the targeted ion pair for the counteranion swap, organic solvents with variable polarity were used, such as CH_3_OH, CH_3_CN and the dipolar nonhydroxylic solvent mixture CH_3_CN:CH_2_Cl_2_ (3:7) and the anion exchange was equally successful with both lipophilic cations and anions.

## 1. Introduction

Besides their recognized value as an alternative to conventional solvents, ionic liquids (ILs) are becoming increasingly useful in a widening range of fields in chemistry leaning toward biology. Indeed, ILs have featured extensively in recent scientific open literature and patents, which reflects their importance in research and development (R&D) [1,2,3,4,5,6,7,8,9]. The greenness of commonly used IL syntheses and purification procedures has been analyzed and evaluated [10] as well as their environmental acceptability and their role in sustainable development [11]. Simple imidazolium quaternary salts with a low melting point are a long-standing IL family and at the same time imidazolium-based systems have continued their progress in anion recognition chemistry and *N*-heterocyclic carbenes (NHCs) [12].

Chemical aspects of imidazolium-based ILs dealing with their preparation, counteranion exchange and purity have been the subject of numerous studies and are currently being investigated with the aim of obtaining pure IL salts, especially halide-free ion pair compounds [4,10,12,13,14,15,16]. A widespread synthesis of imidazolium ILs makes use of a subclass of the Menschutkin reaction, a nucleophilic substitution carried out under neutral conditions between *N*-substituted imidazoles and an alkyl or benzylhalides, affording the targeted imidazolium system in which the counteranion, that is, the halide ion, can be exchanged by different methods. The most frequent method is the classical halide ion exchange with an inorganic salt (MA) that is also used to remove halide ions in ILs. The halide-containing byproduct salts can then be removed by extraction or precipitation followed by filtration. The challenging issue of purification can be addressed by several IL clean-up protocols to eliminate the unwanted halide and/or metal species, among other byproducts [13,14,15,16]. The isolation and purification of pure heteroaromatic quaternary systems can be troublesome, especially if the different ionic species present in the solution-phase have a similar solubility. In this context, a comparative study of the transformation of *N*-azolylpyridinium salts to the corresponding pyridinium azolate betaines showed that the method of choice makes use of a strongly basic anion exchange resin, AER (OH^−^ form) [17]. From 1986 onwards, the AER (OH^−^ form) method has been applied to a variety of *N*-azolylimidazolium and *N*-azolylpyridinium salts with several interanular linkers. Exploiting our standard AER (OH^−^ form) method, the halide-to-anion exchange of different types of bis(imidazolium) cyclophanes, protophanes and calix [4] arenes was carried out using a column chromatography packed with a strongly basic AER (OH^−^ form) followed by immediate collection of the eluates in diluted aqueous acid solution [12,18,19,20,21,22].

The few examples of anion exchange resin application to ILs reported in the open literature use: (a) the AER (OH^−^ form) method, involving the swap of halides for OH^−^, and then to the **[IL][OH]** aqueous or hydroalcoholic solution was slowly added a slight excess of an aqueous acid solution and displacement of the OH^−^ anion by the selected A^−^ anion; or (b) the AER (A^−^ form) method, involving the incorporation of the anion in the resin (OH^−^ form) before the anion is exchanged in ILs. Taking advantage of the AER (OH^−^ form) method, Ohno and co-workers prepared Bio-ILs using strong basic Amberlite (OH^−^ form) to exchange a halide ion for OH^−^, and organic acids or natural aminoacids were added to the aqueous solution of **[IL][OH]** to prepare examples of imidazolium-based **[IL][A]** [23,24]. Choline cations were similarly transformed to the corresponding ionic liquids [25]. In the same way, several ionic liquid buffers were prepared by treatment of the aqueous solution of **[IL][OH]** with organic acids [26]. There are only a few reports exploiting the AER (A^−^ form) method in water or aqueous methanol. Thus, several examples of non-aqueous ionic liquids (NAILs) have been prepared using an AER (PO_4_^3−^ form) [27]. An AER (OH^−^ form) was loaded with mesylate or tosylate anions by treatment with the corresponding sulfonic acid and the prepared AER (R/Ar-SO_3_^−^ form) was then used to transform several *N*,*N*’-dialkylpyrrolidinium iodides to the corresponding sulfonate cations [28]. Loading the anion exchanger with camphorsulfonate anion, AER (CS^−^ form) gave the corresponding **[IL][CS]**from either **[IL][OTs]** [29] or **[IL]Br** [30], the latter following a worthless protocol. Treatment of **[bmim]Cl** with the AER (A^−^ form) -acetate, lactate and nitrate- produced the anion exchange giving **[bmim][A]** [31]. Recently, we examined the preparation of an AER (A^−^ form) conveniently loaded with a selected anion by treatment with either acids or ammonium salts in water or hydroalcoholic media. The anion exchange was carried out in methanol, providing a pure ionic liquid in quantitative yield. This simple procedure not only offers a convenient way to replace halide anions by a broad range of anions in ILs, including task-specific and chiral ILs, but also eliminates halide impurities [32]. Further studies have been directed towards expanding the scope of the halide-for-anion swap in non-aqueous media to representative imidazolium ILs and known examples of bis(imidazolium)-based frameworks for anion recognition. Both lipophylic imidazolium systems and low hydrophilic anions proceeded in excellent to quantitative yields [33].

**Figure 1:**
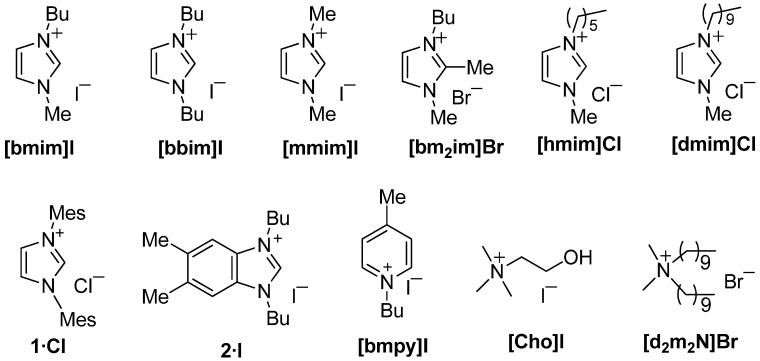
The AER (A^−^ form) method applied to representative quaternary heteroaromatic salts and quaternary ammonium salts.

In this paper we report how the AER (A^−^ form) method can be exploited for a halide-to-anion exchange in several illustrative examples from IL families. The anion source and solvent selection for loading the AER (OH^−^ form) were first examined using different acids or ammonium salts and organic solvent mixtures with variable polarity. The halide-to-anion exchange was then studied using imidazolium-based ILs, random examples of quaternary azolium and pyridinium salts as well as quaternary ammonium salts from the APIs family (Figure 1).

## 2. Results and Discussion

### 2.1. AER (A^−^ Form) Method. Anion Loading

**Scheme 1:**
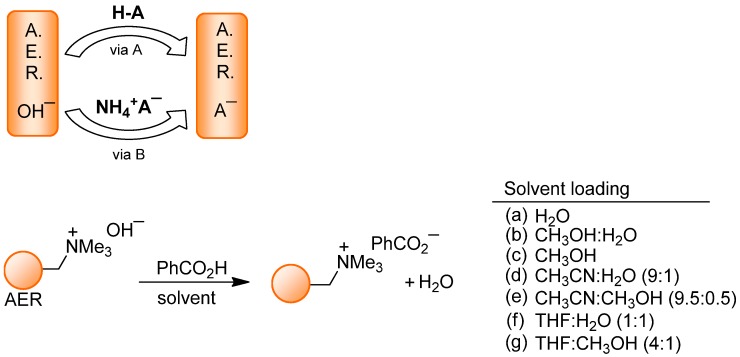
AER (A^−^ form) method: The loading.

*Anion source.* Two methods were used to load the anions: Via *A*, from acids, or via *B*, involving the corresponding ammonium salt (Scheme 1 and Table 1).

The AER (OH^−^ form) was packed in a column and treated with an aqueous or hydromethanolic solution of the acid or ammonium salt. The loading effectiveness was then checked by passing a methanolic solution of **[bmim]I** through the AER column loaded with the target anion and the halide ion to another anion exchange proceeded in quantitative yield. 

**Table 1:** Loading AER (OH^−^ form): Anion source and solvents.

Anion	Source	Solvent	Anion	Source	Solvent
AcO^−^	NH_4_^+^AcO^−^	(a)	AcO^−^	AcOH	(b)
Cl^−^	NH_4_^+^Cl^−^	(a)	Cl^−^	HCl	(a), (b)
PF_6_^−^	NH_4_^+^PF_6_^−^	(a)	PF_6_^−^	HPF_6_	(b)
BF_4_^−^	NH_4_^+^BF_4_^−^	(a)	BF_4_^−^	HBF_4_	(b)
CF_3_SO_3_^−^	NH_4_^+^CF_3_SO_3_^−^	(a)	BzO^−^	BzOH	(b)−(g)
SCN^−^	NH_4_^+^ SCN^−^	(a)	(*S*)-Lactate^−^	(*S*)-Lactic acid	(b)
F¯	NH_4_^+^F^−^	(a)	MeSO_3_^−^	MeSO_3_H	(b)
H_2_PO_4_^−^	NH_4_^+^H_2_PO_4_^−^	(a)	Bu_2_PO_4_^−^	Bu_2_PO_4_H	(b), (c)
HSO_4_^−^	NH_4_^+^HSO_4_^−^	(a)	ClO_4_^−^	HClO_4_	(a), (b)
Ph_4_B^−^	NH_4_^+^Ph_4_B^−^	(d), (e)	NO_3_^−^	HNO_3_	(a), (b)
			Ibu^−^	Ibuprofene	(d), (e)

*Solvent*: (a) H_2_O; (b) CH_3_OH:H_2_O; (c) CH_3_OH; (d) CH_3_CN:H_2_O (9:1); (e) CH_3_CN:CH_3_OH (9.5:0.5); (f) THF:H_2_O (1:1); (g) THF:CH_3_OH (4:1).

Thus, following via *A*, the resin was charged with organic oxoanions derived from carboxilate (R-CO_2_^−^), including chiral (*S*)-lactate, sulfonate (MeSO_3_^−^) or phosphate (Bu_2_PO_4_^−^), together with inorganic anions such as Cl^−^, NO_3_^−^ or ClO_4_^−^, by treatment with the corresponding 1% aqueous acidic solutions. When the loading was performed with the aqueous solution of CF_3_SO_3_H, HF, H_3_PO_4_ or H_2_SO_4_, the polymeric matrix was partially denaturalized by overheating. For this reason, anions such as CF_3_SO_3_^−^, F^−^, H_2_PO_4_^−^ or HSO_4_^−^ were loaded in the resin using aqueous solutions of their ammonium salts (via *B*). In order to confirm the efficiency of the method, both procedures were used to load AcO^−^, Cl^−^, PF_6_^−^ or BF_4_^−^ anions, and identical results were obtained. A few attempts to load anions from their corresponding Na^+^, K^+^ or Li^+^ salt showed, however, that the replacement of OH^−^ in the AER was incomplete, as evidenced by an observed mixture of anions in the checking, and this was not further studied. 

*Solvent selection.* We extended our studies to the loading of hydrophobic anions, and explored alternative solvents and solvent mixtures. Benzoic acid was selected to prepare the AER (BzO^−^ from) and then a methanolic solution of **[bmim]I** was used to check the iodide-to-benzoate anion switch. The resin was first packed in a column and generously washed with the solvent, which was used afterwards to load the benzoate anion. Pure solvents such as distilled CH_3_OH, CH_3_CN, THF and CH_2_Cl_2_ were assayed, but only CH_3_OH provided the optimal loading. Then, several solvent mixtures containing CH_3_CN or THF with H_2_O or CH_3_OH were applied. Among the successful loading solvent mixtures that provided the AER in the BzO^−^ form, those with the lowest proportions of water or methanol were CH_3_CN:H_2_O (9:1), CH_3_CN:CH_3_OH (9.5:0.5), THF:H_2_O (1:1) or THF:CH_3_OH (4:1) (Scheme 1 and Table 1).

These results indicated that a non-aqueous mixture can be used to incorporate lipophylic anions, although the presence of a protic solvent was necessary for the OH^−^ replacement in the AER. Once the suitable solvent conditions were found, acetonitrile solvent mixtures were used to load representative hydrophobic anions: The anti-inflammatory acid ibuprofen to explore via A and ammonium tetraphenylborate to explore via B.

In order to check the loading effectiveness, a methanolic solution of **[bmim]I** was passed through the AER (Ibu^−^ form) or AER (Ph_4_B^−^ form) and the pure **[bmim][Ibu]** [34] or **[bmim][Ph_4_B]** [35] was obtained (see later). These results confirmed that lipophylic anions replace the OH^−^ anion in resin when using the appropriate solvent and the corresponding AER (A¯ form) obtained can then be used for the halide-to-anion switch.

*Loading and exchange ability*. The anion amount that the AER can load and the amount of halide that can then be exchanged were examined. Thus, 2.5 g (~3 cm^3^) of commercial wet A-26 (OH form) was treated with a 1% NH_4_AcO aqueous solution until the pH value of the eluates indicated that loading was complete. Thus, 14.54 mmol of AcO^−^ was loaded with a maximum loading of 5.8 mmol of AcO^−^ per 1 g of this AER. In this context, the synthesis and characterization of resin-supported organotrifluoroborates have recently been reported and the loading was quantified by a UV/Vis spectroscopic analysis [36].

**Scheme 2:**
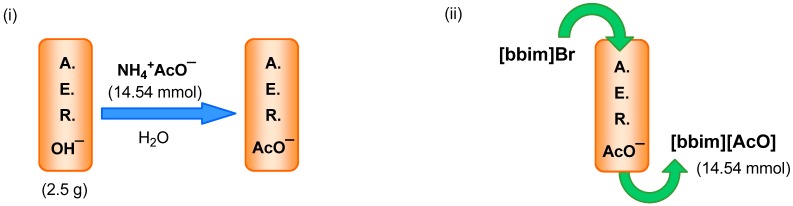
AER (A^−^ form) method. (**i**) Maximum anion loading. (**ii**) Checking anion exchange capacity.

A 50 mM methanolic solution of **[bbim]Br** was passed through the packed column and aliquots were collected periodically and examined by ^1^H-NMR. The related integration of signals corresponding to the anion and imidazolium cation indicated that the exchange process was quantitative up to nearly 14.54 mmol of ionic liquid, suggesting that the Br^−^ exchange could take place as long as there was enough AcO^−^ anion (Scheme 2).

Additionally, it should also be considered that the AER used in the exchange can be recycled by treatment with 10% NaOH aqueous solution, and the recovered AER (OH^−^ form) can be re-utilized for a new anion loading. In the present study, the chosen resin was Amberlyst A-26, given that it allows the use of aqueous mixtures and non-aqueous solvents, but other similar strongly basic anion exchange resins can be used instead.

### 2.2. AER (A^−^ Form) Method. Anion Exchange

**Scheme 3:**
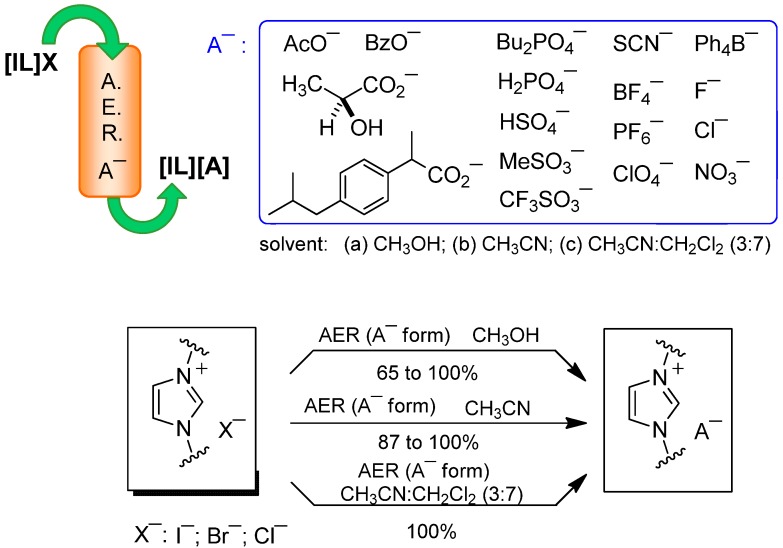
AER (A^−^ form) method applied to imidazolium-based ILs.

Having achieved the loading of several anions in the AER, we examined their efficiency in the counterion exchange in imidazolium-based ILs, including **[bmim]I** or **Br**, **[bbim]I** or **Br** or **[mmim]I** as well as **[bm_2_im]Br**. Thus, a methanolic solution of IL was passed through a column packed with the AER (A^−^ form) previously prepared, and the solvent was removed from the collected eluates. Following this simple method, in almost all cases I^−^ or Br^−^≥ 95% halide-for-anion swapping was obtained except for the hydrophobic anions Ph_4_B^−^ and Ibu^−^, which gave for example, from **[bmim]I** in 65% and 95% yield, respectively (Table 2 and Scheme 3).

**Table 2:** Results of the iodide or bromide exchange in imidazolium ionic liquids.

		[bmim]I or Br		[bbim]I or Br		[mmim]I		[bm_2_im]Br	
Anion	Solvent	Yield (%) ^a^	I^−^ (ppm) ^b^	Yield (%) ^a^	I^−^ (ppm) ^b^	Yield (%) ^a^	I^−^ (ppm) ^b^	Yield (%) ^a^	Br^−^ (ppm) ^b^
AcO^−^	CH_3_OH	100	<20	100	<20	100	<20	98	<13
BzO^−^	CH_3_OH	100	<20	100	<20	95	<20	100	<13
(*S*)-Lactate^−^	CH_3_OH	100	20–40	100	<20	100	<20	100	<13
MeSO_3_^−^	CH_3_OH	100	<20	100	<20	95	<20	92	<13
MeSO_3_^− ^	CH_3_CN	―		―		―		100	<13
Bu_2_PO_4_^−^	CH_3_OH	100	<20	100	<20	100	<20	100	<13
F^−^	CH_3_OH	82	ND ^c^	100	ND ^c^	―		―	
Cl^−^	CH_3_OH	100	ND	100	ND	100	ND	―	
PF_6_^−^	CH_3_OH	100	20–40	100	<20	100	<20	91	ND
PF_6_^−^	CH_3_CN	―		―		―		100	13–26
NO_3_^−^	CH_3_OH	100	<20	100	<20	100	20–40	―	
ClO_4_^−^	CH_3_OH	100	100–120	100	20–40	100	20–40	―	
BF_4_^−^	CH_3_OH	100	<20	100	<20	100	20–40	97	13–26
H_2_PO_4_^−^	CH_3_OH	100	<20	100	20–40	100	<20	―	
HSO_4_^−^	CH_3_OH	100	<20	100	20–40	100	<20	―	
CF_3_SO_3_^−^	CH_3_OH	100	<20	100	<20	100	<20	100	<13
SCN^−^	CH_3_OH	100	ND	100	ND	100	ND	100	ND
Ph_4_B^−^	CH_3_OH	65	<20	45	<20	―		―	
Ph_4_B^− ^	CH_3_CN	95	<20	100	<20	―		91	<13
Ibu^−^	CH_3_OH	95	<20	―		―		―	
Ibu^−^	CH_3_CN	100	<20	―		―		96	<13

ND: Not Determined. ^a^ Recovered new ion pair. Yields ≥95% in CH_3_OH were not further examined in CH_3_CN; ^b^ Halide contents after anion exchange determined by the silver chromate test; ^c^ Analyzed by HPLC/IC from exchange of Br^−^ by F^−^: Presence of Br^−^ anion was not observed.

Moreover, no evidence of *N*-heterocyclic carbenes (NHCs) and/or dealkylation by-product formation was observed despite the basic environment, e.g., anion basicity [13,37,38]. The purity of the ionic liquids obtained by this method was qualitatively determined using ^1^H-NMR spectra, and/or ESI(−)-MS experiments, and according to the silver chromate test, most analyses indicated low halide contents (<20 ppm for I^−^ or <13 ppm for Br^−^). Further quantification of possible halide impurity was restricted by instrumental limitation [32].

Although the halide exchange occurred with lipophylic anions such as Ph_4_B^−^, when the process was carried out in methanol the yield of the recovered compound decreased to 65%, due to the change of solubility of the new ion pair, which caused their partial retention in the resin. Hence, organic solvents such as CH_3_CN or CH_2_Cl_2_ or CH_3_CN:CH_2_Cl_2_ solvent mixtures were then selected to perform the halide switch, the treatment of **[bmim]I** with AER (BzO^−^ form) being used to check the process. The results indicated that the exchange was successful in both aprotic organic solvents, while the use of pure CH_2_Cl_2 _as a solvent in our usual exchange procedure was discarded due to experimental difficulties, after testing several combinations, the mixture with the highest proportion of dichloromethane that was workable was found to be CH_3_CN:CH_2_Cl_2_ (3:7). This enabled a quantitative iodide-for-benzoate swap and afforded the possibility for those exchanges of hydrophobic ionic species.

**Scheme 4:**
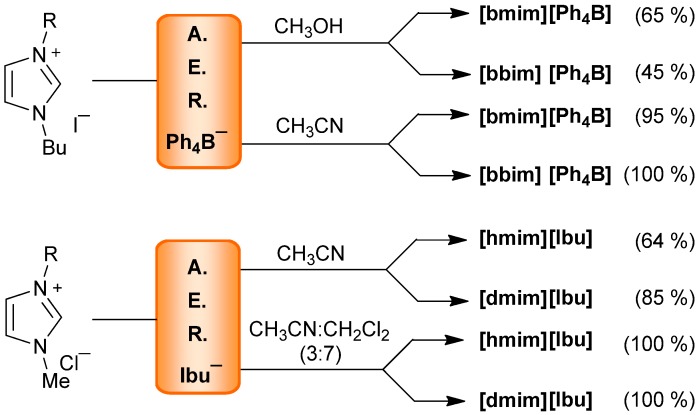
AER (A^−^ form) method. Halide to lipophylic anion exchange.

Accordingly, the preparation of **[bmim][Ph_4_B]** or **[bbim][Ph_4_B]** from their corresponding iodide salts using the AER (Ph_4_B^¯^ form) in CH_3_OH provided the corresponding ion pair in 65% and 45% yield, respectively. The yield increased to 95% and 100% when CH_3_CN was used, confirming that less polar solvents in the exchange process substantially improved the recovery of the less hydrophilic ion pair (Scheme 4). Similarly, **[bm_2_im]Br** was directly studied in CH_3_CN and the exchange of Ph_4_B^−^ and Ibu^−^ anions proceeded in 91% and 96% yields, respectively (Table 2).

Hydrophobic salts such as hexylmethylimidazolium chloride **[hmim]Cl** or decylmethylimidazolium chloride **[dmim]Cl** were used to swap the chloride for the ibuprofenate anion. A solution of the corresponding ionic liquid in CH_3_CN was passed through the AER (Ibu^−^ form) affording the anion exchange in ≤95% yields. A more lipophylic solvent was then used and quantitative results were obtained with the dipolar nonhydroxylic organic solvent mixture CH_3_CN:CH_2_Cl_2_ (3:7) (Scheme 4 and Table 3).

**Table 3:** Comparative results of chloride exchange in **[hmim]Cl** and **[dmim]Cl**.

Cation	Anion	Solvent	Yield (%) ^a^	Cl^−^ (ppm) ^b^
hmim	Ibu^−^	CH_3_CN	90	<6
	Ibu^−^	CH_3_CN:CH_2_Cl_2_ (3:7)	100	<6
dmim	Ibu^−^	CH_3_CN	87	<6
	Ibu^−^	CH_3_CN:CH_2_Cl_2_ (3:7)	100	<6

^a^ Yield of the recovered new ion pair; ^b^ Halide contents after anion exchange determined by silver chromate test.

**Scheme 5:**
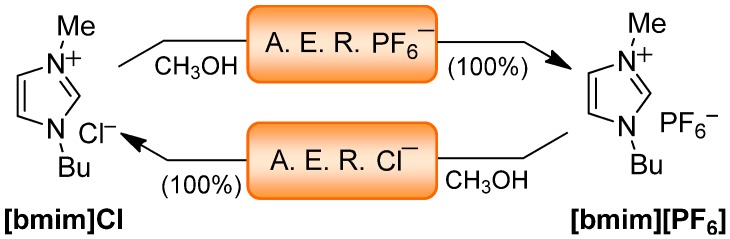
AER (A^−^ form) method: Chloride to hexafluorophosphate exchange and vice versa.

Next, the AER (A^−^ form) method was extended to other anions. Thus, a methanolic solution of **[bmim]Cl** was passed through the AER (PF_6_^−^ form) packed column and the eluates were analyzed after the solvent was removed. The ^1^H-NMR spectrum coincided with that of **[bmim][PF_6_]**, which indicated a successful exchange confirmed by the silver chromate test (<6 ppm of Cl^−^). Similarly, a methanolic solution of **[bmim][PF_6_]** was passed through the AER (Cl^−^ form) packed column and the ^1^H-NMR spectrum also showed the quantitative exchange (Scheme 5). Thus, a conveniently loaded AER can be used to carry out the swapping from a range of anions other than halides. The process was followed by ^1^H-NMR, since the signal corresponding to the C(2)-H of the imidazolium moiety is generally the most influenced by the nature of the anion (see Experimental section); for example, the chemical shift value measured in CDCl_3_ (0.02 M) is 9.07 ppm in **[bmim][PF_6_]** while in the same conditions this value is 10.99 ppm in **[bmim]Cl**.

**Scheme 6:**
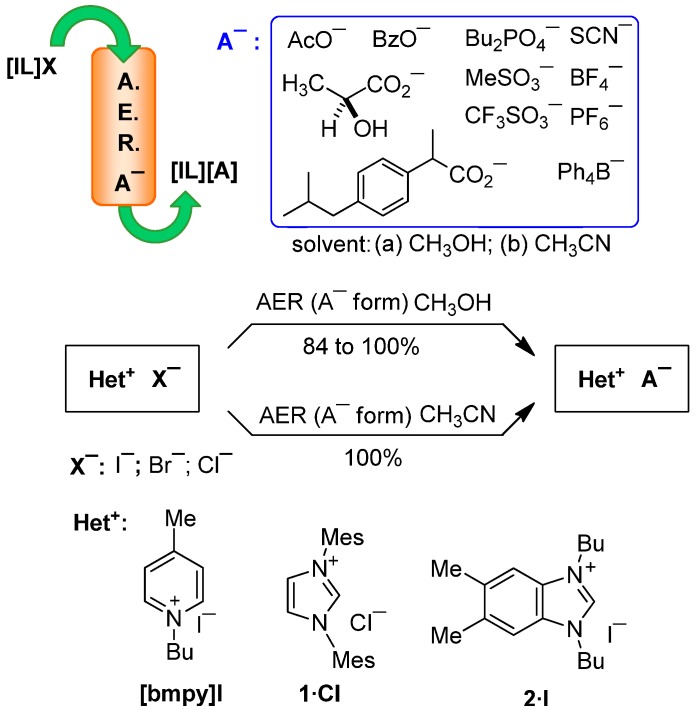
Halide-to-anion exchange in quaternary azolium and pyridinium salts.

Regarding other heteroaromatic cationic systems, pyridinium (**[bmpy]I**) or benzimidazolium (**2·I**) nuclei were chosen as examples to carry out the anion swap, together with the well known NHC precursor 1,3-dimesitylimidazolium salt (**1·Cl**) (Figure 1 and Scheme 6). A methanolic solution of **[bmpy]I** was passed through a column packed with the convenient AER (A^−^ form), and the corresponding pure **[bmpy][A]** were obtained in ≥ 98% yield, except for the acetate anion, which was recovered in 84% yield. Changing to a more hydrophobic solvent, the iodide-for-acetate swap in acetonitrile proceeded in quantitative yield. In the treatment of **[bmpy]I** with the AER (A^−^ form), there was no evidence in any case of the formation of decomposition byproducts, despite the basicity of some anions, e.g., acetate (Table 4).

Following the same procedure, a methanolic solution of the new benzimidazolium salt **2·I** was used to obtain the corresponding ion pair **2·A**, with excellent yields. The iodide exchange of the white solid **2·I** (m.p. 150–1 °C) led to oily ion pairs at room temperature or solids with a low melting point (see Experimental section). The new benzimidazolium salts **2·A** are related to previously reported benzimidazolium salts with potential use as new materials, e.g., ionic liquid crystals [39] and crystalline metal-containing ILs [40,41,42]. Likewise, the chloride anion in 1,3-dimesitylimidazolium salt **1·Cl** can be successfully displaced by a wide range of anions using the AER (A^−^ form). When the swapping took place in methanol, the recovery of **1·A** was between 80 to 95%, but in acetonitrile yields were nearly quantitative (Table 4). In all cases the silver chromate test revealed the low chloride content after the exchange (<6 ppm), which confirmed the easy swapping of Cl^−^ anion. These examples demonstrated that the method is also effective with non IL cationic systems, and is a general method for preparing tuneable quaternary heteroaromatic salts. Accordingly, the well-known catalyst precursor **1·Cl** [43] was easily transformed in **1·A**, and the presence of different counteranions could potentially modulate the formation of organometallic complexes due to their improved solubility and the stabilizing effect of anion participation.

**Table 4:** Results of the halide exchange in pyridinium, benzimidazolium and imidazolium salts **[bmpy][I]**, **1·Cl** and **2·I**.

		[bmpy][I]		1·Cl		2·I	
Anion	Solvent	Yield (%) ^a^	I^−^ (ppm) ^b^	Yield (%) ^a^	Cl^−^ (ppm) ^b^	Yield (%) ^a^	I^−^ (ppm) ^b^
AcO^−^	CH_3_OH	84	<20	95	<6	100	<20
AcO^−^	CH_3_CN	100	<20	―		―	
BzO^−^	CH_3_OH	100	<20	92	<6	95	<20
BzO^−^	CH_3_CN	―		100	<6	―	
(*S*)-Lactate^−^	CH_3_OH	100	<20	98	<6	100	<20
MeSO_3_^−^	CH_3_OH	100	<20	91	<6	90	<20
MeSO_3_^−^	CH_3_CN	―		100	<6	100	
Bu_2_PO_4_^−^	CH_3_OH	100	<20	95	<6	97	<20
PF_6_^−^	CH_3_OH	100	<20	100	<6	100	<20
BF_4_^−^	CH_3_OH	98	<40	79	<6	100	<20
BF_4_^−^	CH_3_CN	―		100	<6	―	
CF_3_SO_3_^−^	CH_3_OH	100	<20	88	<6	95	<20
CF_3_SO_3_^−^	CH_3_CN	―		95	<6	―	
SCN^−^	CH_3_OH	100	ND	91	ND	100	ND
SCN^−^	CH_3_CN	―		97	ND	―	
Ph_4_B^−^	CH_3_CN	―		82	<6	―	

ND: Not Determined. ^a^ Yield of the recovered new ion pair. Yields ≥95% in CH_3_OH were not further examined in CH_3_CN; ^b^ Halide contents after anion exchange determined by silver chromate test.

Two examples of quaternary ammonium salts were selected from the API family to confirm the efficiency of the method with this type of ILs. The choline lactate (**[Cho][Lact]**) [44] was quantitatively prepared from the corresponding **[Cho]I** using the AER (Lact^−^ form) in methanol. Didecyldimethylammonium bromide (**[d_2_m_2_N]Br**) was transformed to the antibacterial-anti-inflammatory didecyldimethylammonium ibuprofenate **[d_2_m_2_N][Ibu]** [45].

**Scheme 7:**
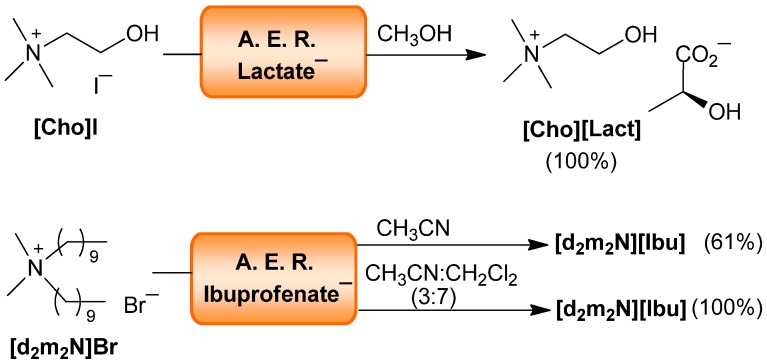
AER (A^−^ form) method. Quaternary ammonium salts.

This hydrophobic ammonium salt required the lipophylic solvent mixture CH_3_CN:CH_2_Cl_2_ (3:7) to afford the quantitatively iodide-to-ibuprofenate switch, since in acetonitrile the yield was only 61% (Scheme 7 and Table 5).

**Table 5:** The halide exchange in quaternary ammonium salts **[Cho]I** and **[d_2_m_2_N]Br**.

Cation	Anion	Solvent	Yield (%) ^a^	I^−^ (ppm) ^b^
Cho	(*S*)-Lactate^−^	CH_3_OH	100	<20
d_2_m_2_N	Ibu^−^	CH_3_CN	61	<13
	Ibu^−^	CH_3_CN: CH_2_Cl_2_ (3:7)	100	<13

^a^ Yield of the recovered new ion pair; ^b^ Halide contents after anion exchange determined by silver chromate test.

The quaternary heteroaromatic and ammonium ILs prepared taking advantage of the AER (A^−^ form) method in organic solvents were characterized by ^1^H-NMR, electrospray ionization mass spectrometry in the negative mode and the halide content was determined by the silver chromate test. When the recovery of the new ion pair **[IL][A]** was ≤95%, a new assay was performed using a less polar organic solvent, which improved the yield in the range of 95% to 100%. As mentioned above, the use of an anion exchange resin implies the possibility of sorbet contamination [13,46], so, nano-particulates may be an issue to analyze. The analysis of possible nano-particulate contamination was, however, beyond the scope of the present study.

Recapping the results, the AER (A^−^ form) method applied to different examples of quaternary heteroaromatic salts and ionic liquids permitted the halide to be swapped for assorted anions in excellent yields of ≥95% when the appropriate organic solvent or solvent mixture was used. It was confirmed that the AER (A^−^ form) method is efficient with imidazolium-based ILs, improving the currently operative procedures of classical counteranion exchange. Against a large pool of quaternary heteroaromatic and ammonium salts, we limited ourselves to the eleven examples shown in Figure 1 to validate the AER (A^−^ form) method in non-aqueous media. 

## 3. Experimental

### 3.1. General

Ion exchanger resin Amberlyst A-26 (Aldrich, OH^−^ form), **[hmim]Cl**, **[dmim]Cl**, **[Cho]I** and **[d_2_m_2_N]Br** together with all acids, ammonium salts, reagents and solvents were purchased from commercial suppliers, unless mentioned otherwise, and used without further purification. All solvents were reagent grade and methanol and THF were distilled prior to use. **[bmim]I** [32], **[bmim]Br** [32], **[bbim]I** [32], **[bbim]Br** [32], **[mmim]I** [32], **[bm_2_im]Br** [47], **[bmpy]I** [48], and **1·Cl** [49] were prepared according with the literature. ^1^H-NMR and ^13^C-NMR spectra were recorded on a Varian Gemini 300 (300 MHz for ^1^H and 75.4 MHz for ^13^C) spectrometer at 298 K. ^1^H and ^13^C chemical shifts were referenced with TMS as an internal reference. Mass spectrometric analyses were performed on a LC/MSD-TOF (2006) mass spectrometer with a pumping system HPLC Agilent 1100 from Agilent Technologies at Serveis Científico-Tècnics of universitat de Barcelona. The pH was measured with benchmeter pH1100 (Eutech Instrunments, Nijkerk, The Netherlands), using Hamilton Flushtrode pH electrode for hydroalcoholic solutions.

### 3.2. Loading the AER (OH^−^ Form) with Acids or Ammonium Salts

A glass column (1 cm diameter ) packed with 2.5 g (~3 cm^3^) of commercial wet strongly basic anion exchange Amberlyst A-26 (OH^−^ form) was washed with water, and the column bed was equilibrated progressively with water-solvent mixtures until reaching the selected solvent media used afterwards for anion loading (~25 mL of each solvent mixture). A 1% acid or ammonium salt solution in the appropriate solvent was passed slowly through the resin until the eluates had the same pH value as the original selected acid solution, and then the resin was washed generously with solvent until constant pH. The process was carried out at room temperature, using gravity as the driving force.

### 3.3. Anion Exchange: General Procedure

A solution of the imidazolium salt (0.5–0.6 mmol) in 10 mL of the selected solvent was passed slowly through a column packed with ~3 cm^3^ of Amberlyst A-26 (A^–^ form), and then washed with 25 mL of solvent. The combined eluates were evaporated, and the residue obtained was dried in a vacuum oven at 60 °C with P_2_O_5_ and KOH pellets.

### 3.4. Silver Chromate Test

The amount of halide contents was determined by a silver chromate test following a similar protocol to that described by Sheldon and co-workers [31]. An aqueous solution (5 mL) of potassium chromate (5% p/v in Milli-Q water, 0.257 M) was added to the sample (5–10 mg). To 1 mL of the resulting dark yellow solution was added a minimum amount of silver nitrate aqueous solution (0.24% p/v in Milli-Q water, 0.014 M). A persistent red suspension of silver chromate would be observed if the sample was free of halide. The minimum measurable amount of silver nitrate aqueous solution was 0.011 mL; consequently, the detection limit is approx. 6 ppm for Cl^−^, 13 ppm for Br^−^ or 20 ppm for I^−^. The halide content was determined at least twice for each sample.

### 3.5. 1,3-Dibutyl-5,6-dimethylbenzimidazolium Iodide(**2·I**)

A suspension of 5,6-dimethyl-1*H*-benzimidazole (1.00 g, 6.84 mmol) and NaH (0.40 g, 16.66 mmol) in dry THF (100 mL) was stirred under argon atmosphere at 60 °C for 1 h, and then 1-iodobutane (1.50 g, 8.15 mmol) was added. The reaction mixture was stirred at 65 °C for 48 h, and then 5 mL of ethanol were added. The solvent was evaporated to dryness, and the residue was treated with water (50 mL) and extracted with CH_2_Cl_2_ (3 × 50 mL). The organic solution was dried over anhydrous Na_2_SO_4_, filtered and the solvent was eliminated under vacuum. A mixture of the previous yellow oil (1.34 g, 6.62 mmol) and 1-iodobutane (1.23 g, 6.70 mmol) was stirred under argon atmosphere at 85 °C for 20 h. The reaction mixture was washed with dry diethyl ether (3 × 25 mL) in an ultrasonic bath, providing the pure **2·I** as a white solid (2.47 g, 93% yield). M.p. 150–1 °C. δ_H_ (300 MHz; CDCl_3_; Me_4_Si) 0.99 (6H, t, *J* = 7.4 Hz, N-C_3_H_6_-*CH*_3_), 1.45 (4H, m, N-C_2_H_4_-*CH*_2_-CH_3_), 2.02 (4H, m, N-CH_2_-*CH*_2_-C_2_H_5_), 2.47 (6H, s, C(5,6)-Me), 4.56 (4H, t, *J* = 7.4 Hz, N-*CH_2_*-C_3_H_7_), 7.42 (2H, s, C(4,7)-H) and 11.01 (1H, s, C(2)-H). δ_C_ (75.4 MHz, CDCl_3_) 13.5, 19.8, 20.7, 31.3, 47.3, 112.8, 129.8, 137.5, 140.4. HRMS-ESI(+) Calcd for C_17_H_27_N_2_ [M]^+^ 259.2169, found 259.2167.

*Melting points of compounds ***2·A**: **2·MeSO_3_**, 62–3 °C; **2**·Bu_2_PO_4_, 56–7 °C; **2·PF_6_**, 85–6 °C; **2·BF_4_**, 109–110 °C; **2·CF_3_SO_3_**, 78–9 °C; **2·SCN**, 64–5 °C; **2·AcO**, **2·BzO** and **2·Lact** are oily compounds at room temperature

### 3.6. 1H-NMR Data of Compounds [bmim][A] (Table 6), [bbim][A] (Table 7), [mmim][A] (Table 8), [hmim][A] (Table 9), [dmim][A] (Table 9), [bm2im][A] (Table 10), [bmpy][A] (Table 11), 1·A (Table 12), 2·A (Table 13), [Cho][A] (Table 14) and [d2m2N][A] (Table 14)

**Table 6:** ^1^H-NMR chemical shift values of 1-butyl-3-methylimidazolium salt **[bmim][A]** (300 MHz) at 298 K ^a^. 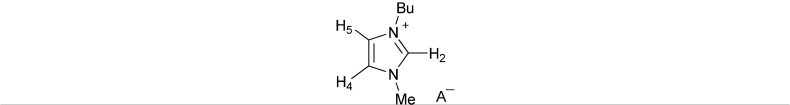

Anion	Solvent	H-2	H-4	H-5	Bu	Me	A^−^
AcO^−^	CDCl_3_	11.35	7.09	7.08	4.30; 1.86; 1.37; 0.96	4.06	1.99
BzO^−^	CDCl_3_	11.00	7.09	7.09	4.29; 1.84; 1.33; 0.92	4.08	8.10; 7.33
( *S*)-Lactate^−^	CDCl_3_	11.19	7.17	7.17	4.31; 1.89; 1.38; 0.98	4.08	3.46; 1.41
MeSO_3_^−^	CDCl_3_	10.21	7.25	7.20	4.28; 1.87; 1.38; 0.97	4.05	2.80
Bu_2_PO_4_^−^	CDCl_3_	10.19	7.36	7.23	4.25; 1.80; 1.33; 0.88	4.00	3.80;1.54;1.33; 0.88
I^−^^b^	CDCl_3_	10.27	7.52	7.44	4.35; 1.93; 1.41; 0.99	4.14	
Br^−^	CDCl_3_	10.41	7.46	7.37	4.35; 1.91; 1.40; 0.98	4.13	
F^−^	CDCl_3_	(c)	7.50	7.33	4.29; 1.87; 1.36; 0.95	4.06	
Cl^−^	CDCl_3_	10.99	7.31	7.24	4.33; 1.91; 1.40; 0.98	4.13	
PF_6_^−^	CDCl_3_	9.07	7.26	7.23	4.20; 1.88; 1.38; 0.97	3.98	
NO_3_^−^	CDCl_3_	10.02	7.35	7.30	4.25; 1.88; 1.38; 0.97	4.02	
ClO_4_^−^	CDCl_3_	9.15	7.30	7.26	4.23; 1.89; 1.39; 0.98	4.02	
BF_4_^−^	CDCl_3_	8.98	7.28	7.24	4.21; 1.87; 1.39; 0.97	3.98	
CF_3_SO_3_^−^	CDCl_3_	9.27	7.32	7.28	4.21; 1.88; 1.38; 0.97	3.99	
SCN^−^	CDCl_3_	9.59	7.36	7.31	4.32; 1.92; 1.41; 0.99	4.11	
Ibu^−^	CDCl_3_	9.86	7.10	7.02	4.02; 1.66; 1.24; 0.87	3.71	7.26; 6.95; 3.53; 2.35; 1.75; 1.39; 0.82
AcO^−^	CD_3_CN	9.25	7.35	7.32	4.14; 1.80; 1.31; 0.93	3.84	1.66
BzO^−^	CD_3_CN	9.43	7.29	7.28	4.19; 1.80; 1.30; 0.92	3.86	7.93; 7.27
MeSO_3_^−^	CD_3_CN	8.63	7.37	7.34	4.16; 1.80; 1.31; 0.94	3.83	2.43
I^−^	CD_3_CN	8.56	7.39	7.35	4.14; 1.81; 1.31; 0.94	3.83	
Cl^−^	CD_3_CN	9.04	7.39	7.36	4.15; 1.80; 1.31; 0.93	3.84	
PF_6_^−^	CD_3_CN	8.42	7.35	7.31	4.11; 1.79; 1.30; 0.93	3.80	
NO_3_^−^	CD_3_CN	8.58	7.37	7.34	4.13; 1.81; 1.31; 0.94	3.82	
ClO_4_^−^	CD_3_CN	8.43	7.37	7.35	4.12; 1.81; 1.32; 0.94	3.82	
BF_4_^−^	CD_3_CN	8.43	7.36	7.33	4.12; 1.82; 1.32; 0.94	3.81	
CF_3_SO_3_^−^	CD_3_CN	8.43	7.36	7.33	4.12; 1.80; 1.32; 0.94	3.81	
SCN^−^	CD_3_CN	8.49	7.37	7.34	4.13; 1.80; 1.30; 0.94	3.82	
Ph_4_B^−^	CDCl_3_	4.54	6.01	5.84	3.16; 1.33; 1.13; 0.89	2.76	7.52; 6.97; 6.78
Ph_4_B^−^	CD_3_CN	8.19	7.27 ^d^	7.27 ^d^	4.05; 1.77; 1.30; 0.93	3.74	7.27; 6.99; 6.84
Ph_4_B^−^	DMSO-d_6_	9.06	7.74	7.67	4.13; 1.75; 1.24; 0.89	3.82	7.16; 6.91; 6.78

^a^ Solution concentrations are 0.02 M; ^b^ Unambiguous assignments were made by NOESY-1D (400 MHz); ^c^ Signal not observed; ^d^ Included in the phenyl signal.

**Table 7:** ^1^H-NMR chemical shift values of 1,3-dibutylimidazolium salt **[bbim][A]** (300 MHz) at 298 K ^a^. 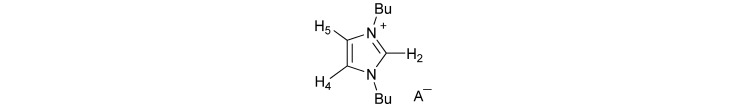

Anion	Solvent	H-2	H-4,5	Bu	A^−^
AcO^−^	CDCl_3_	11.32	7.14	4.35; 1.86; 1.39; 0.97	2.01
BzO^−^	CDCl_3_	11.40	7.16	4.34; 1.87; 1.35; 0.93	8.10; 7.32
(*S*)-Lactate^−^	CDCl_3_	11.29	7.14	4.33; 1.87; 1.37; 0.96	4.02; 1.39
MeSO_3_^−^	CDCl_3_	9.73	7.51	4.30; 1.88; 1.37; 0.96	2.75
Bu_2_PO_4_^−^	CDCl_3_	11.05	7.11	4.37; 1.88; 1.40; 0.94	3.87; 1.62; 1.40; 0.94
I^−^	CDCl_3_	10.34	7.38	4.38; 1.95; 1.42; 0.99	
Br^−^	CDCl_3_	10.58	7.42	4.36; 1.90; 1.37; 0.95	
F^−^	CDCl_3_	(b)	7.17	4.30; 1.89; 1.40; 0.98	
Cl^−^	CDCl_3_	11.05	7.23	4.38; 1.92; 1.41; 0.98	
PF_6_^−^	CDCl_3_	9.05	7.23	4.24; 1.88; 1.39; 0.98	
NO_3_^−^	CDCl_3_	9.89	7.39	4.25; 1.86; 1.33; 0.94	
ClO_4_^−^	CDCl_3_	9.24	7.38	4.26; 1.88; 1.37. 0.96	
BF_4_^−^	CDCl_3_	9.12	7.36	4.23; 1.87; 1.36; 0.95	
H_2_PO_4_^−^	CDCl_3_	10.59	7.31	4.40; 1.84; 1.34; 0.92	
HSO_4_^−^	CD_3_CN	10.84	7.40	4.39; 1.84; 1.34; 0.91	
CF_3_SO_3_^−^	CDCl_3_	9.49	7.28	4.26; 1.88; 1.38; 0.98	
SCN^−^	CDCl_3_	9.18	7.34	4.25; 1.88; 1.38; 0.97	
Ph_4_B^−^	CDCl_3_	(b)	5.81	3.10; 1.30; 1.13; 0.89	7.50; 6.98; 6.82
Ph_4_B^−^	DMSO-d_6_	9.19	7.79	4.15; 1.77; 1.26; 0.90	7.18; 6.92; 6.78

^a^ Solution concentrations are 0.02 M. ^b^ Signal not observed.

**Table 8:** ^1^H-NMR chemical shift values of 1,3-dimethylimidazolium salt **[mmim][A]** (300 MHz) at 298 K ^a^. 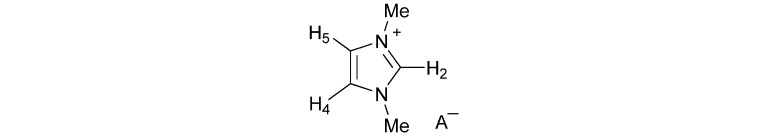

Anion	Solvent	H-2	H-4,5	Me	A^−^
AcO^−^	CD_3_CN	9.05	7.32	3.83	1.69
BzO^−^	CD_3_CN	9.29	7.33	3.85	7.93; 7.28
(*S*)-Lactate^−^	CDCl_3_	11.04	7.15	4.03	3.80; 1.38
MeSO_3_^−^	CD_3_CN	8.58	7.33	3.83	2.43
Bu_2_PO_4_^− ^	CDCl_3_	10.88	7.15	4.04	3.86; 1.61; 1.39; 0.90
I^−^	CD_3_CN	8.48	7.34	3.83	
Cl^−^	CD_3_CN	8.57	7.34	3.83	
PF_6_^−^	CD_3_CN	8.38	7.32	3.82	
NO_3_^−^	CD_3_CN	8.57	7.34	3.83	
ClO_4_^−^	CD_3_CN	8.45	7.33	3.82	
BF_4_^−^	CD_3_CN	8.43	7.33	3.82	
H_2_PO_4_^− ^	CDCl_3_	10.26	7.30	4.09	
HSO_4_^−^	CDCl_3_	10.19	7.34	4.09	
CF_3_SO_3_^−^	CD_3_CN	8.45	7.33	3.82	
SCN^−^	CD_3_CN	8.44	7.33	3.83	

^a^ Solution concentrations are 0.02 M.

**Table 9:** ^1^H-NMR chemical shift values of imidazolium salts **[hmim][A]** and **[dmim][A]** in CDCl_3_ (300 MHz) at 298 K ^a,b^. 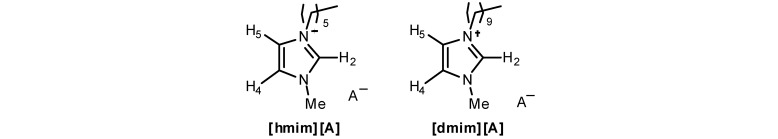

Cation	Anion	H-2	H-4	H-5	C_n_H_n+1_	Me	A^−^
hmim	Cl^−^	10.80	7.44	7.31	4.30; 1.89; 1.30; 0.86	4.11	–
	Ibu^−^	9.72	7.08	7.01	4.05; 1.74; 1.26; 0.86	3.75	7.28; 7.01; 3.54;2.37; 1.78; 1.41; 0.86
dmim	Cl^−^	10.82	7.38	7.27	4.32; 1.89; 1.27; 0.86	4.12	–
	Ibu^−^	10.58	7.01	6.99	4.11; 1.78; 1.25; 0.87	3.81	7.31; 6.98; 3.60;2.39; 1.79; 1.46; 0.87

^a^ Solution concentrations are in the range of 0.015 to 0.025 M; ^b^ H-4 and H-5 assignments were made according **[bmim]I**.

**Table 10:** ^1^H-NMR chemical shift values of 1-butyl-2,3-dimethylimidazolium salt **[bm_2_im][A]** in CDCl_3_ (300 MHz) at 298 K ^a^. 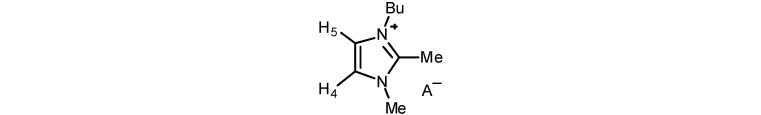

Anion	H-4	H-5	Me-2	Me-3	Bu	A^−^
AcO^−^	7.58	7.36	2.59	3.82	4.06; 1.67; 1.26; 0.86	1.72
BzO^−^	7.54	7.27	2.50	3.71	3.90; 1.58; 1.23; 0.85	7.97; 7.27
(S)-Lactate^−^	7.49	7.26	2.70	3.92	4.12; 1.79; 1.40; 0.98	3.87; 1.30
MeSO_3_^−^	7.47	7.27	2.69	3.94	4.14; 1.80; 1.38; 0.98	2.74
Bu_2_PO_4_^−^	7.55	7.27	2.68	3.92	4.13; 1.76; 1.37; 0.96	3.77; 1.56; 1.37; 0.89
Br^−b^	7.76	7.56	2.83	4.04	4.24; 1.81; 1.40; 0.98	
I^−^	7.60	7.46	2.80	3.98	4.18; 1.80; 1.39; 0.94	
PF_6_^−^	7.46	7.30	2.70	3.90	4.11; 1.79; 1.40; 0.96	
BF_4_^−^	7.40	7.27	2.68	3.88	4.10; 1.79; 1.40; 0.97	
CF_3_SO_3_^−^	7.32	7.22	2.66	3.86	4.09; 1.80; 1.40; 0.97	
NCS^−^	7.43	7.32	2.77	3.96	4.17; 1.83; 1.43; 0.98	
Ph_4_B^−^	6.38	6.28	2.39	2.98	3.36; 1.52; 1.25; 0.92	7.46; 6.99; 6.83
Ph_4_B^−c^	7.63	7.60	2.56	3.73	4.09; 1.68; 1.29; 0.90	7.17; 6.92; 6.78
Ibu^−^	7.30	7.07	2.37	3.57	3.88; 1.56; 1.22; 0.85	7.23; 6.94; 3.45; 2.33; 1.73; 1.33; 0.81

^a^ Solution concentrations are 0.02 M; ^b^ Unambiguous assignments were made by NOESY-1D (400 MHz); ^c^ In DMSO-d_6_.

**Table 11:** ^1^H-NMR chemical shift values of 1-butyl-4-methylpyridinium salt **[bmpy][A]** in CDCl_3_ (300 MHz) at 298 K ^a^. 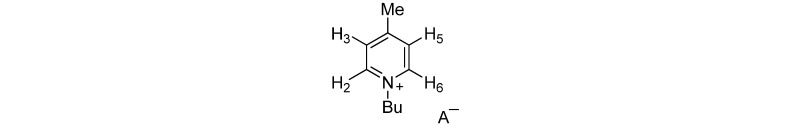

Anion	H-2,6	H-3,5	Me	Bu	A^−^
AcO^−^	9.35	7.82	2.62	4.82; 1.96; 1.35; 0.94	1.96
BzO^−^	8.94	7.70	2.47	4.67; 1.82; 1.25; 0.83	8.00; 7.31
(S)-Lactate^−^	9.05	7.81	2.57	4.65; 1.88; 1.35; 0.87	3.89; 1.26
MeSO_3_^−^	9.09	7.83	2.57	4.65; 1.91; 1.32; 0.87	2.68
Bu_2_PO_4_^−^	9.36	7.83	2.53	4.72; 1.89; 1.30; 0.83	3.78; 1.50; 1.30; 0.83
I^−^	9.24	7.90	2.66	4.84; 2.00; 1.41; 0.95	
PF_6_^−^	8.60	7.80	2.66	4.54; 1.95; 1.39; 0.95	
BF_4_^−^	8.73	7.82	2.66	4.60; 1.95; 1.39; 0.95	
CF_3_SO_3_^−^	8.80	7.82	2.65	4.60; 1.94; 1.38; 0.94	
NCS^−^	8.94	7.91	2.70	4.77; 2.03; 1.44; 0.99	

^a^ Solution concentrations are 0.02 M.

**Table 12:** ^1^H-NMR chemical shift values of 1,3-bis(mesityl)imidazolium salt **1·A** in CDCl_3_ (300 MHz) at 298 K ^a^. 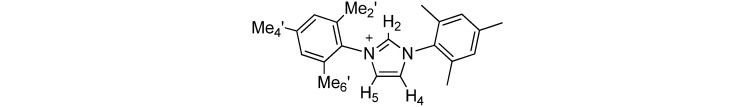

Anion	H-2		H-4,5		Me-2',6'		Me-4'		H-3'		A^−^	
AcO^−^	11.54		7.46		2.20		2.35		7.04		2.16	
BzO^−^	11.03		7.44		2.07		2.25		6.87		7.63; 7.14	
(S)-lactate^−^	10.31		7.56		2.10		2.32		7.00		3.65; 1.04	
MeSO_3_^−^	9.83		7.63		2.09		2.31		6.98		2.31	
Bu_2_PO_4_^−^	10.76		7.67		2.12		2.30		6.97		3.43; 1.32; 1.20; 0.79	
Cl^−^	10.98		7.57		2.20		2.34		7.03			
PF_6_^−^	8.77		7.57		2.14		2.37		7.07			
BF_4_^−^	9.19		7.57		2.09		2.32		6.99			
CF_3_SO_3_^−^	9.29		7.57		2.09		2.34		7.01			
SCN^−^	9.70	7.63		2.19		2.37		7.08				
Ph_4_B^−^	6.32	7.06		2.02		2.20		6.77		7.30; 6.88; 6.77		
Ph_4_B^−b^	9.64	8.25		2.11		2.35		7.20		7.18; 6.92; 6.78		

^a^ Solution concentrations are in the range of 0.01 to 0.02 M; ^b^ In DMSO-d_6_.

**Table 13:** ^1^H-NMR chemical shift values of 1,3-dibutyl-5,6-dimethylbenzimidazolium salt **2·A** in CDCl_3_ (300 MHz) at 298 K ^a^. 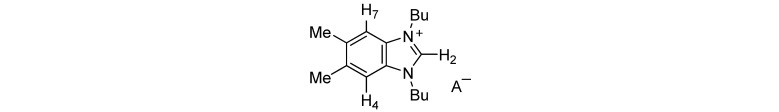

Anion	H-2	H-4,7	Me	Bu	A^−^
AcO^−^	11.86	7.37	2.46	4.55; 1.96; 1.42; 0.97	2.03
BzO^−^	11.91	7.37	2.45	4.56; 2.00; 1.41; 0.93	8.11; 7.34
(S)-lactate^−^	11.39	7.36	2.43	4.49; 1.92; 1.37; 0.93	4.03; 1.37
MeSO_3_^−^	10.63	7.40	2.47	4.53; 1.98; 1.44; 0.99	2.84
Bu_2_PO_4_^−^	11.52	7.36	2.45	4.57; 1.96; 1.41; 0.97	3.90; 1.62; 1.41; 0.90
I^−^	10.98	7.43	2.46	4.55; 2.02; 1.46; 0.99	
PF_6_^−^	9.25	7.43	2.48	4.41; 1.97; 1.43; 0.99	
BF_4_^−^	9.33	7.48	2.45	4.43; 1.94; 1.40; 0.94	
CF_3_SO_3_^−^	9.86	7.42	2.47	4.48; 1.97; 1.43; 0.98	
SCN^−^	10.13	7.43	2.48	4.53; 2.02; 1.47; 1.00	

^a^ Solution concentrations are 0.02 M.

**Table 14:** ^1^H-NMR chemical shift values of quaternary ammonium salts **[Cho][A]** and [**d_2_m_2_N][A]** (300 MHz) at 298 K. 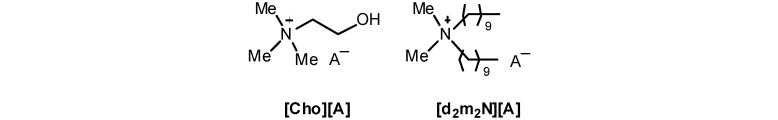

Cation	Anion	Solvent	Me	N^+^-CH_2_-CH_2_-OH	A^−^
Cho	I^−^	CD_3_CN	3.12	3.95; 3.41; 3.59(OH)	–
	( *S*)-Lactate^−^	CD_3_CN	3.13	3.95; 3.43; 3.67(OH)	3.78; 1.19
				N^+^-C_n_H_n+1_	
d_2_m_2_N	Br^−^	CDCl_3_	3.41	3.51; 1.65; 1.30; 0.88	–
	Ibu^−^	CDCl_3_	3.01	3.10; 1.52; 1.26; 0.88	7.30; 7.00; 3.57;2.39; 1.81; 1.42; 0.88

## 4. Conclusions

Faced with a large variety of quaternary imidazolium and ammonium salts, the present study using an anion exchange resin (A^−^ form) in non-aqueous media was based on a choice of eleven examples taken from the IL pool **[IL]X** that could serve to evaluate the halide-for-anion swap. Significant aspects of the reported AER (A^−^ form) process are: (i) the anion loading of the AER (OH^−^ form) with acids and ammonium salts in solvent mixtures of different polarities according to the hydrophobicity of the anion source; (ii) the anion exchange using the AER (A^−^ form) method in organic solvents was easily applied to several imidazolium, benzimidazolium, pyridinium and ammonium salts, the halide-for-anion exchange progressing in excellent to quantitative yields. Depending on the hydrophobic nature of the targeted organic salts, the counteranion exchange was accomplished in organic solvents of variable polarity and dipolar nonhydroxylic organic solvent mixtures ranging from the lowest proportions of water or methanol to lipophylic solvent mixtures such as CH_3_CN:CH_2_Cl_2_ (3:7). 

On the whole, the AER (A^−^ form) method in organic solvents is a method of choice for exchanging the halide anions for a variety of anions in quaternary heteroaromatic and ammonium salts, simultaneously removing halide impurities, which is often a troublesome task. This anion exchange method could be adapted to a wide array of charged molecules crucial to advances in interdisciplinary fields in chemistry.
